# Glutaminase 2 expression is associated with regional heterogeneity of 5-aminolevulinic acid fluorescence in glioblastoma

**DOI:** 10.1038/s41598-017-12557-3

**Published:** 2017-09-22

**Authors:** Sojin Kim, Ja Eun Kim, Yong Hwy Kim, Taeyoung Hwang, Sung Kwon Kim, Wen Jun Xu, Jong-Yeon Shin, Jong-Il Kim, Hyoungseon Choi, Hee Chan Kim, Hye Rim Cho, Anna Choi, Tamrin Chowdhury, Youngbeom Seo, Yun-Sik Dho, Jin Wook Kim, Dong Gyu Kim, Sung-Hye Park, Hyeonjin Kim, Seung Hong Choi, Sunghyouk Park, Se-Hoon Lee, Chul-Kee Park

**Affiliations:** 1 Department of Neurosurgery, Seoul National University College of Medicine, Seoul National University Hospital, Seoul, Korea; 2 Cell and Gene Therapy Products Division, National Institute of Food and Drug Safety Evaluation, Ministry of Food and Drug Safety, Cheongju, Korea; 3 Department of Biomedical Engineering, Johns Hopkins University School of Medicine, Baltimore, MD USA; 4 Department of Neurosurgery, Gyeongsang National University School of Medicine, Gyeongsang National University Hospital, Jinju, Korea; 5 College of Pharmacy, Natural Product Research Institute, Seoul National University, Seoul, Korea; 6 Genomic Medicine Institute, Medical Research Center, Seoul National University, Seoul, Korea; 7 Department of Biochemical and Molecular Biology, Seoul National University College of Medicine, Seoul, Korea; 8 DMC R&D center, Samsung Electronics Co., Ltd., Seoul, Korea; 9 Department of Biomedical Engineering, College of Medicine and Institute of Medical and Biological Engineering, Medical Research Center, Seoul National University, Seoul, Korea; 10 Department of Radiology, Seoul National University College of Medicine, Seoul National University Hospital, Seoul, Korea; 11 Department of Neurosurgery, Yeungnam University College of Medicine, Daegu, Korea; 12 Department of Pathology, Seoul National University College of Medicine, Seoul National University Hospital, Seoul, Korea; 13 Division of Hematology-Oncology, Department of Medicine, Samsung Medical Center, Sungkyunkwan University School of Medicine, Seoul, Korea

## Abstract

Fluorescence-guided surgery using 5-aminolevulinic acid (5-ALA) is now a widely-used modality for glioblastoma (GBM) treatment. However, intratumoral heterogeneity of fluorescence intensity may reflect different onco-metabolic programs. Here, we investigated the metabolic mechanism underlying the heterogeneity of 5-ALA fluorescence in GBM. Using an in-house developed fluorescence quantification system for tumor tissues, we collected 3 types of GBM tissues on the basis of their fluorescence intensity, which was characterized as strong, weak, and none. Expression profiling by RNA-sequencing revealed 77 genes with a proportional relationship and 509 genes with an inverse relationship between gene expression and fluorescence intensity. Functional analysis and *in vitro* experiments confirmed *glutaminase 2* (*GLS2*) as a key gene associated with the fluorescence heterogeneity. Subsequent metabolite profiling discovered that insufficient NADPH due to *GLS2* underexpression was responsible for the delayed metabolism of 5-ALA and accumulation of protoporphyrin IX (PpIX) in the high fluorescence area. The expression level of GLS2 and related NADPH production capacity is associated with the regional heterogeneity of 5-ALA fluorescence in GBM.

## Introduction

Glioblastoma (GBM) is one of the most devastating cancers of the brain; the current best practice of multimodal treatment results in an overall survival rate of 27.2% in 2 years^1^. However, in the modern era of GBM management, it is now generally accepted that the extent of surgical resection significantly affects patient prognosis^2^. Diverse efforts for the improvement of the resection rate in GBM have recently been attempted that integrate the newest technologies, including fluorescence-guided surgery (FGS), intraoperative neurophysiological monitoring, intraoperative magnetic resonance imaging, and neuronavigation/ultrasound-assisted surgery^3^. Among them, FGS using 5-aminoleveulinic acid (5-ALA) has rapidly become a widely accepted practice for treatment of high-grade gliomas, owing to its ability to improve the extent of resection while preserving the patient’s functional status^4–6^. 5-ALA is a metabolic targeting agent and the natural precursor of the fluorescent protoporphyrin IX (PpIX) in the heme biosynthesis pathway^7^. The temporary accumulation of PpIX by overloaded exogenous 5-ALA emits enhanced red fluorescence (wavelength 600–720 nm) selectively in cancer cells under a blue light source (wavelength: 375–440 nm), thus enabling the identification of cancer boundaries otherwise hardly distinguishable with a white light microscope during GBM surgery^8^. The sensitivity and specificity of 5-ALA fluorescence have been reported to be as high as 85% and 75% in GBM^9^.

Despite the widespread clinical use of 5-ALA FGS in GBM and the extensive studies exploring its mechanism of selective fluorescence, the conclusive explanation has not yet been reported. Only sporadic reports on alterations in heme biosynthetic enzymes and porphyrin transporters have been proposed for the mechanism of the preferential accumulation of PpIX in cancer cells, but the evidences were inconsistent among the tumor types^10^. Among the suggested heme biosynthetic enzymes with changes in the expression or activity in cancer tissues, only *ferrochelatase* (*FECH*) has been found to be downregulated in GBM tissues compared with the normal brain^11^. However, there is no evidence of mutation in the *FECH* gene itself in GBM, thus suggesting that another indirect regulatory mechanism is responsible for the decreased FECH activity that is yet to be discovered. Among the altered porphyrin transporters in cancer cells, ATP-binding cassette sub-family B member 6 (ABCB6) shows higher expression in glioma tissue than in normal brain tissue and is correlated with 5-ALA/PpIX fluorescence^12^. However, the non-specific location of ABCB6 identified in cell organelles suggests that the porphyrin transport can be bidirectional, thus resulting in its weak contribution to intracellular PpIX accumulation^13–15^. No direct evidence of alterations for other heme biosynthetic enzymes or porphyrin transporters in human glioma tissues has been reported until now.

Considering the nature of the temporary accumulation of PpIX after 5-ALA administration in cancer cells, focusing on metabolic reprogramming in cancer may be a more reasonable way to investigate the mechanism of selective fluorescence. Studies on the mechanism of 5-ALA fluorescence in gliomas from the perspective of metabolomics are scarce. Reprogrammed metabolism is one of the hallmarks of cancer, including GBM^16^. Altered carbon metabolism involving enhanced aerobic glycolysis and a decreased tricarboxylic acid (TCA) cycle is a common feature of proliferating cancer cells^17^. We have previously shown that mutation of isocitrate dehydrogenase 1 (IDH1), one of the TCA cycle isozymes commonly mutated in lower grade gliomas, is related to the enhancement of PpIX accumulation and fluorescence by exogenous 5-ALA in malignant glioma cells^18^. Further evidence of crosstalk between the TCA cycle and the heme biosynthesis pathway in cancer has been demonstrated^19^. However, the onco-metabolomic relationships between the TCA cycle and the heme biosynthesis pathway in the absence of mutations in TCA cycle enzymes have not been elucidated. A novel hypothesis for the metabolic mechanism is needed to explain the selective 5-ALA-induced fluorescence in GBM tissue, because most GBM tissues express wild-type TCA enzymes^20^.

The issue of intratumoral heterogeneity has been proposed and proven in terms of genomics, proteomics and histology in many cancers^21^. With the recent advances in metabolomics, intratumoral metabolic heterogeneity has also emerged in the spotlight^22,23^. Regional heterogeneity of metabolic reprogramming in solid tumors is thought to be linked to various microenvironments and specific pathways implicated by genomic alterations^22,24^. Glioblastoma is one of the representative models for intratumoral heterogeneity with regard to genomic alterations and microscopic features. The interesting characteristic about 5-ALA fluorescence in GBM is that there is also regional heterogeneity in the fluorescence intensity^25^. The degree of fluorescence intensity in GBM has been reported to be correlated with cell density^25^. However, this intratumoral fluorescence heterogeneity is more likely to be the direct result of intratumoral metabolic heterogeneity. Therefore, the genomic and metabolomic mechanisms of 5-ALA fluorescence in GBM are expected to be determined by investigating the cause of intratumoral fluorescence heterogeneity.

In this study, we have identified *glutaminase 2* (*GLS2)* as a key gene in the regulation of 5-ALA metabolism by transcriptome profiling by using multi-regional GBM samples with different fluorescence intensities. Additionally, *in vitro* experiments validated the relationship between *GLS2* and 5-ALA fluorescence/PpIX accumulation in GBM cell lines, thus demonstrating that insufficient NADPH production, a major determinant of the heterogenous 5-ALA/PpIX fluorescence in correlation with *GLS2* underexpression is a major determinant of the heterogeneous 5-ALA/PpIX fluorescence. On the basis of the observed findings, we proposed a hypothesis for the metabolic mechanism of 5-ALA fluorescence in GBM tissue.

## Results

### Regional heterogeneity of 5-ALA fluorescence in GBM tissue

**Figure 1:**
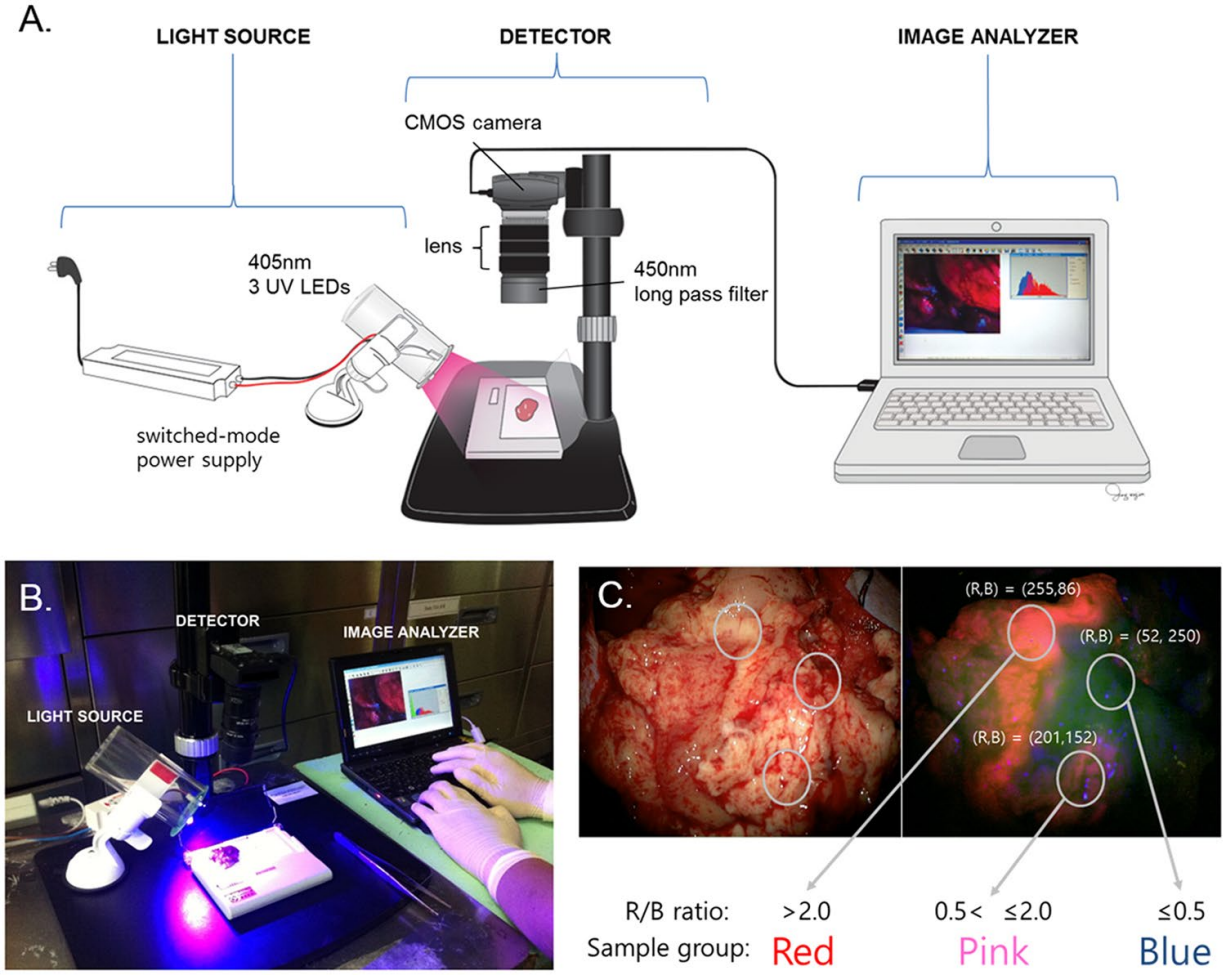
Collection of glioblastoma samples according to the regional heterogeneity of fluorescence intensity. (**A**) Schematic illustration of the fluorescence measurement and quantification system for surgically obtained samples (all copyright of this artwork by Mi-Jin Jung of Biomedical Illustration & Design Company transferred to Nature Publishing Group). (**B**) Real situation of sample management immediately after tumor resection. (**C**) The samples were classified and acquired according to the quantified fluorescence intensity. The fluorescence intensities were measured according to the red/blue (R/B) ratios in the captured images and then were separated into the following 3 classes: strong (red, R/B ratio >2.0), weak (pink, 0.5 < R/B ratio ≤2.0), and none (blue, R/B ratio ≤0.5).

We devised an in-house system of fluorescence quantification in tumor tissues (Fig. 1). The fluorescence intensity was measured as the red/blue (R/B) ratio in captured images immediately after resection of the tumor during 5-ALA FGS. Using this system, we collected 3 classes of GBM tissues in each of 5 patients according to the fluorescence intensity as follows: strong (red, R/B ratio >2.0), weak (pink, 0.5 < R/B ratio ≤2.0), and none (blue, R/B ratio ≤0.5). All 5 patients showed clear intratumoral fluorescence heterogeneity in their GBM tissues (Supplementary Figure 1 and Supplementary Table 1). Individually separated samples were snap-frozen in liquid nitrogen before nucleic acid extraction, and parts of the tissues were sent for histological diagnosis. Each red, pink, and blue area showed different histological grades, IV, III, and II, respectively, in all patients (Supplementary Figure 2).

### Key matched genomics for the regional heterogeneity of fluorescence

**Figure 2:**
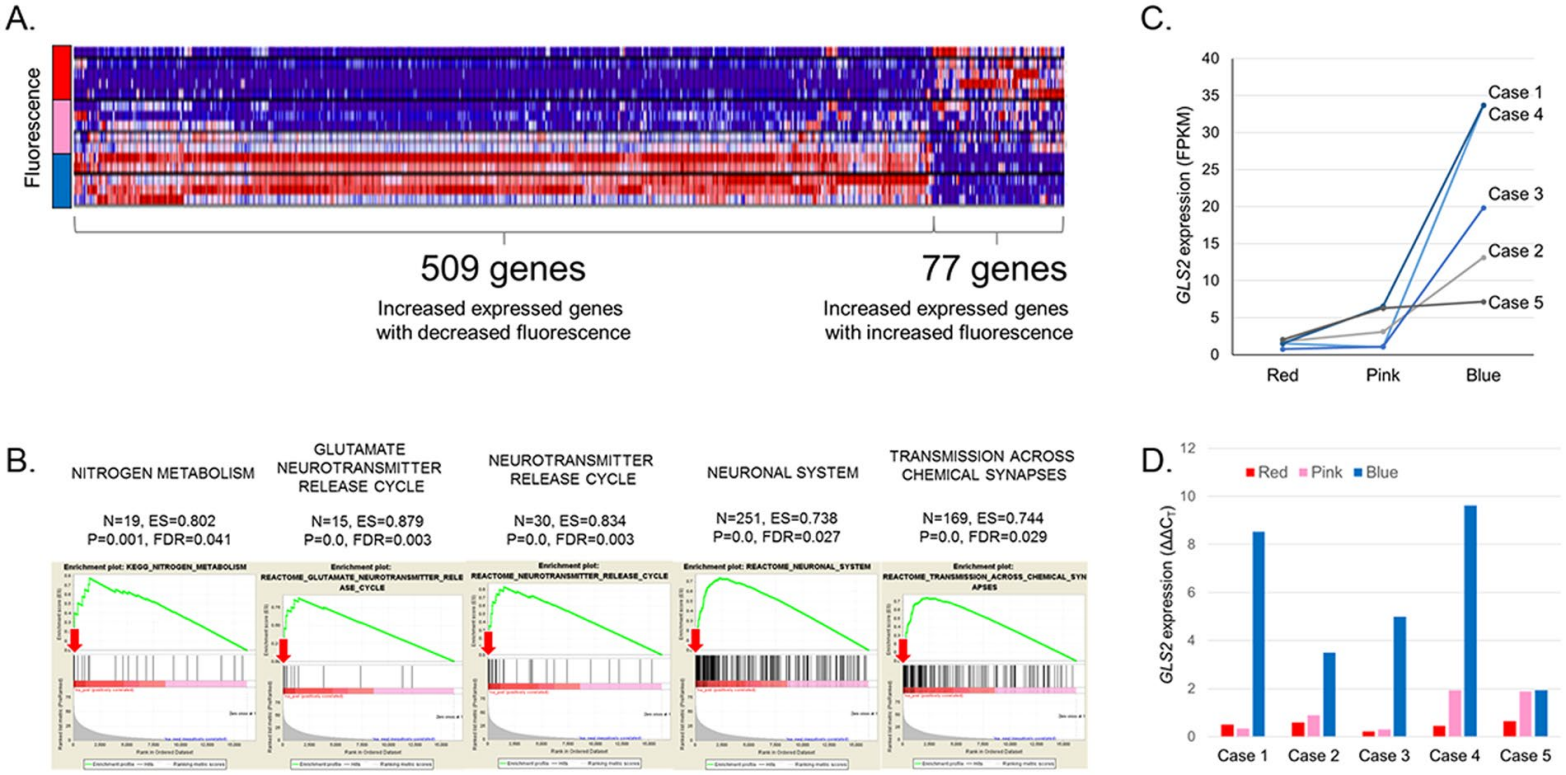
Identification of key genes associated with 5-aminolevulinic acid (5-ALA) fluorescence intensity in glioblastoma. (**A**) Results of RNA-sequencing of classified samples, which were analyzed according to differential expression along with the fluorescence intensity (fold change >2, p < 0.05, FDR < 0.01). Seventy-seven genes with positive correlation and 509 genes with negative correlation between the expression level and fluorescence intensity were identified. (**B**) Pathway analysis using gene set enrichment analysis (GSEA) resulted in 30 significantly enriched gene sets of (p < 0.01, FDR < 0.25). Among them, *GLS2* (red arrows) was repeatedly found with a high enrichment score in the selected gene sets of metabolism and the neuronal system. (**C**) From the results of the RNA-seq FPKM values, *GLS2* was highly expressed in areas of no fluorescence intensity, and the expression was decreased in areas of positive fluorescence in all samples. (**D**) The expression level of *GLS2* in all samples, as measured by quantitative real-time PCR, confirmed the result of RNA-seq.

After the extraction of RNA from samples, RNA-sequencing of total RNA was performed to profile the gene expression levels in each sample. To identify the key genes related to fluorescence intensity, we first sorted out the genes that showed a linear trend of expression levels among the red, pink, and blue sample groups. A total of 3,344 genes were identified to have either an increasing or decreasing trend of expression in the red, pink, and blue groups (FDR < 0.01). Next, the genes were filtered further on the basis of the condition of a clear linear trend among the 3 groups by selecting the differentially expressed genes with fold changes greater than two between groups. The final result elicited 77 genes with positive correlation, and 509 genes with negative correlation between the expression level and fluorescence intensity (Fig. 2A and Supplementary Dataset).

To identify the most plausible key genes for fluorescence heterogeneity, the list of candidate genes was classified according to predefined functional modules by using gene set enrichment analysis (GSEA) after being ranked on the basis of a score calculated from the adjusted p-value. Among the large numbers of gene sets from the GSEA that were enriched positively and negatively, 10 gene sets from the Kyoto Encyclopedia of Genes and Genomes (KEGG)pathway (http://www.genome.jp/kegg/) and 20 gene sets from the REACTOME pathway (http://reactome.org/) were displayed significantly with p-value < 0.01 and FDR < 0.25 (Supplementary Table 2). Because 5-ALA fluorescence is the result of the temporary intracellular accumulation of PpIX in cancer cells, we focused on gene sets related to cell metabolism as well as the neuronal system. Interestingly, *GLS2* was repeatedly found with a high enrichment score in the selected gene sets (Fig. 2B). The expression of *GLS2* was inversely correlated with the fluorescence intensity in fragments per kilobase of transcript per million mapped reads (FPKM) values (Fig. 2C). Additionally, we confirmed the expression level of *GLS2* by quantitative real-time PCR (Fig. 2D). Therefore, it is plausible that *GLS2* is a key candidate gene for heterogenous 5-ALA fluorescence intensity in GBM.

### *GLS2* attenuates 5-ALA fluorescence in GBM cells

**Figure 3:**
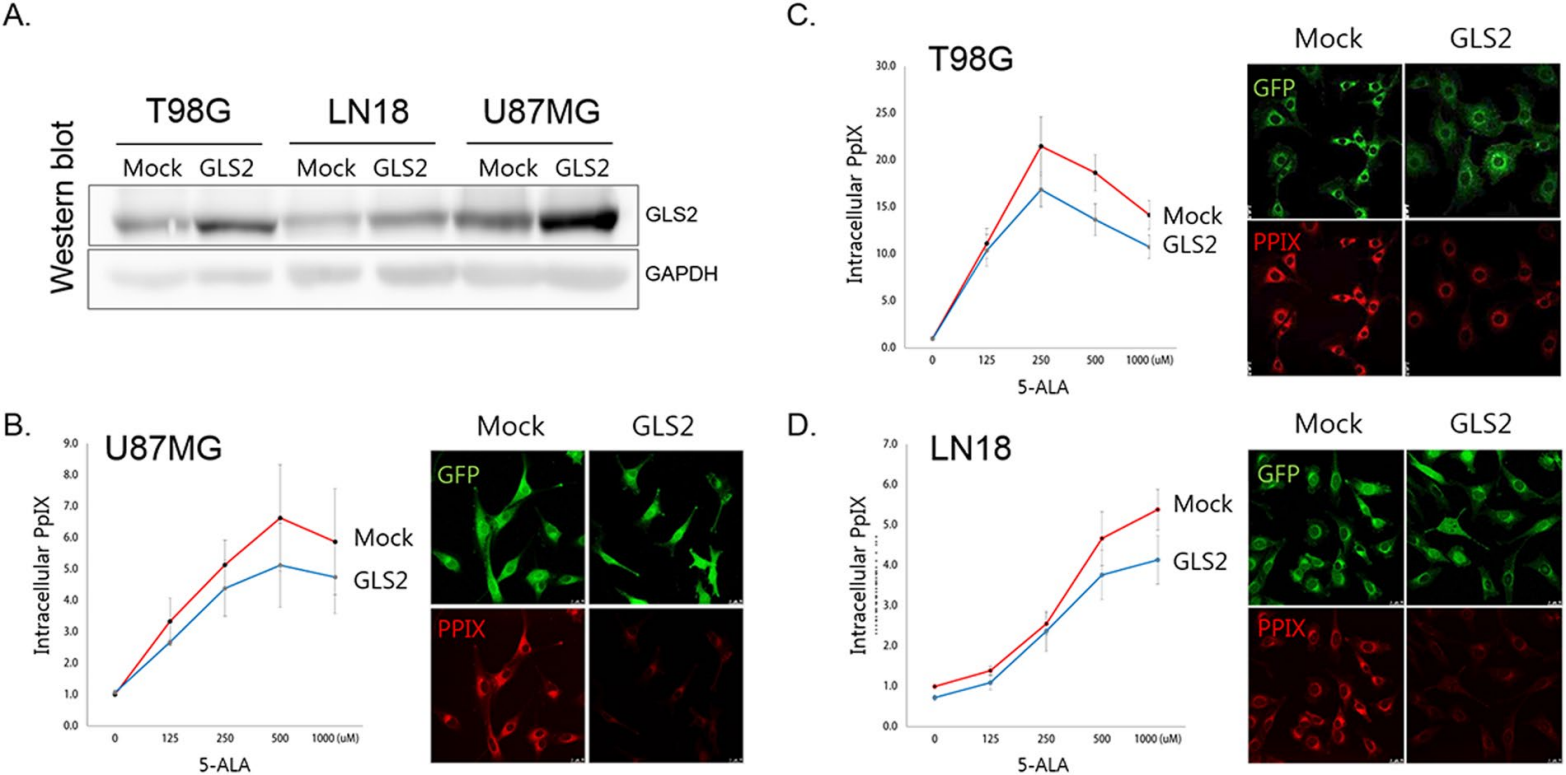
*In vitro* evidence of an association between *GLS2* expression and 5-ALA fluorescence. (**A**) The overexpression of the *GLS2* gene after transduction into three glioblastoma cell lines (T98G, LN18, and U87MG) was confirmed at the protein level. Western blot images are cropped for the GLS2/GAPDH blots and the full-length of gel image is included in Supplementary Figure 3. (**B**–**D**) All cell lines with *GLS2* overexpression showed decreased PpIX accumulation and fluorescence intensity after 5-ALA treatment (p < 0.05, average value of 4 duplicate experiments). The concentration of PpIX after 5-ALA treatment was expressed as relative fluorescence units (RFUs) normalized against total cell protein levels. Confocal laser scanning microscope images were taken 2 hours after 5-ALA treatment. PpIX was visible as red fluorescence and was located mainly in the cytoplasm (green fluorescence protein (GFP)).

To verify the role of *GLS2* in 5-ALA fluorescence in cancer cells, the lentiviral vector containing the *GLS2* gene construct was established and transduced into GBM cell lines (T98G, U87MG, and LN18). *In vitro* measurements of intracellular PpIX after 5-ALA treatment showed a significant decrease (Fig. 3). Fluorescence was evident after 5-ALA treatment in GBM cell lines but was attenuated in cells with *GLS2* transduction (Fig. 3). Therefore, it was confirmed that the underexpression of *GLS2* is associated with increased 5-ALA fluorescence intensity.

### *GLS2* induces NADPH production, and NADPH is consumed during 5-ALA metabolism

**Figure 4:**
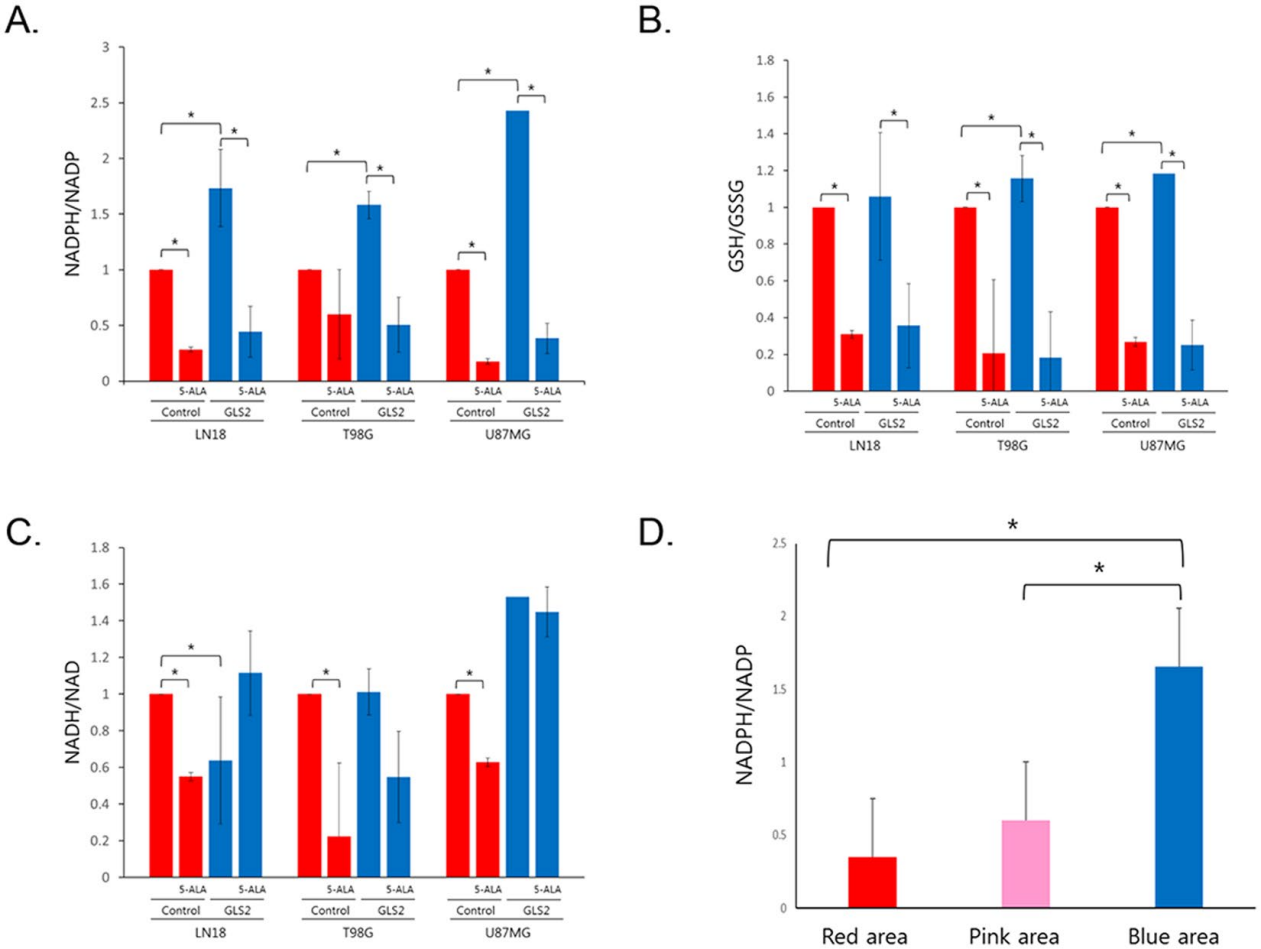
(**A–C**) Metabolite screening by using liquid chromatography-mass spectrometry (LC-MS) on glioblastoma cell lines with or without *GLS2* expression after 5-aminolevulinic acid (5-ALA) treatment. Each sample was tested in triplicate. NADPH/NADP levels were significantly increased with *GLS2* expression, but they decreased rapidly after 5-ALA treatment in all cell lines (**A**). GSH/GSSG levels were also decreased after 5-ALA treatment, but they increased with *GLS2* expression in 2 cell lines with a relatively small amount of change (**B**). NADH/NAD levels were decreased after 5-ALA treatment only in cells without *GLS2* expression (**C**). (**D**) Direct measurement of NADPH/NADP levels in glioblastoma tissues showing different fluorescence intensities (n = 30) confirmed increased NADPH/NADP levels in the no-tumor area without fluorescence.

To screen for metabolic changes that might explain the difference in 5-ALA metabolism between cell lines with and without *GLS2* expression, we profiled the metabolites from the lysates of GBM cell lines with or without *GLS2* expression after 5-ALA exposure, by using liquid chromatography–mass spectrometry (LC-MS). Among the metabolites tested, NADPH production was significantly increased when *GLS2* expression was enhanced, and NADPH was rapidly consumed after the introduction of 5-ALA (Fig. 4A). The ratio of reduced glutathione (GSH) to oxidized glutathione (GSSG) was also slightly increased with *GLS2* expression, and it was decreased after 5-ALA treatment (Fig. 4B). The NADH/NAD ratio was decreased after 5-ALA treatment in cells with *GLS2* underexpression, but changes in the NADH/NAD ratio were not observed in cells with *GLS2* expression with or without 5-ALA treatment (Fig. 4C). There were no intracellular energy metabolism changes regarding *GLS2* expression or 5-ALA treatment, as represented by AMP, ADP, and ATP levels (Supplementary Figure 4). Overall, NADPH/NADP and GSH/GSSG levels were significantly decreased after 5-ALA treatment, and *GLS2* expression induced increased levels of NADPH/NADP, thus possibly increasing the capacity for 5-ALA metabolism.

To confirm the regional differences in the NADPH levels in GBM, the NADPH/NADP levels were measured in 10 paired GBM tissues located in the red, pink, and blue fluorescence areas. The NADPH/NADP level was significantly increased in the area without 5-ALA fluorescence compared with that with positive fluorescence (Fig. 4D).

### Hypothesis for the metabolic mechanism of 5-ALA fluorescence in GBM tissue

**Figure 5:**
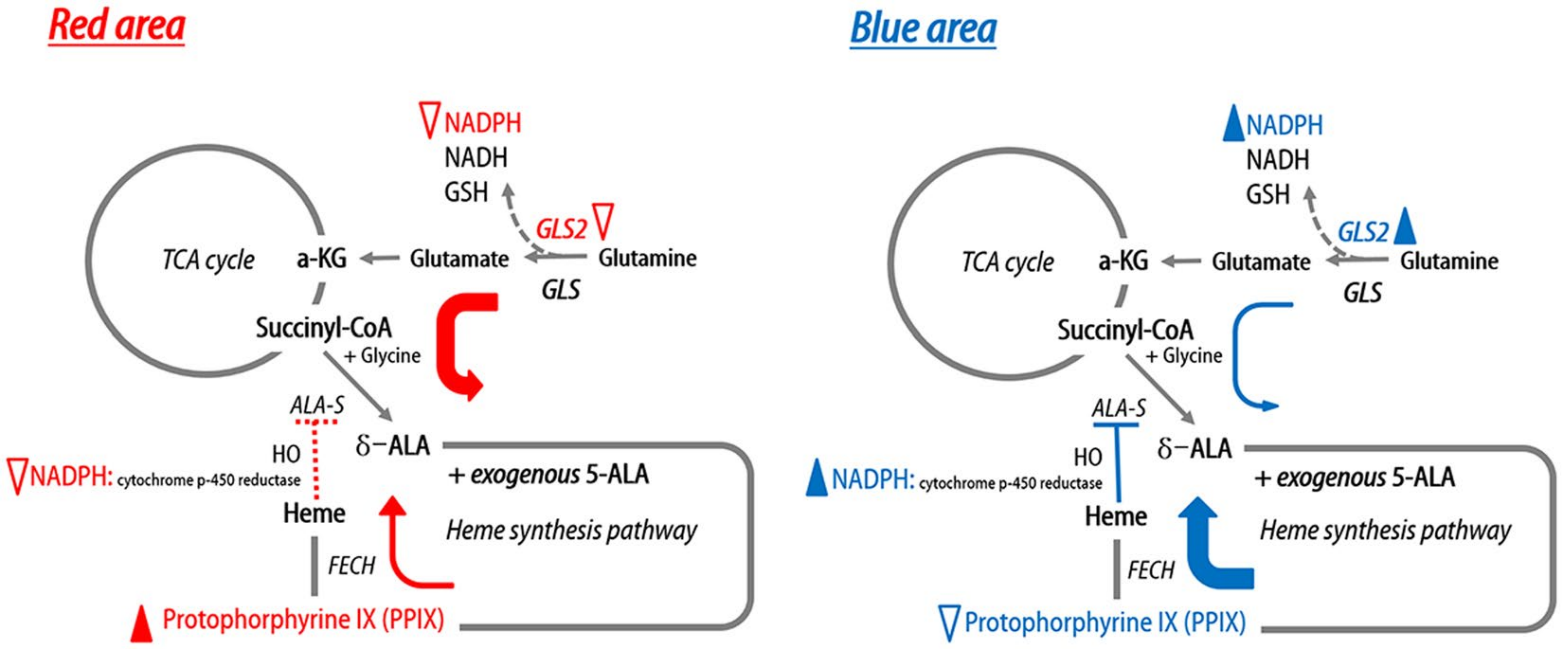
Schematic illustration of the hypothesis for the regional heterogeneity of 5-aminolevulinic acid (5-ALA) fluorescence intensity in glioblastoma. At the area of strong fluorescence intensity (red area), decreased expression of glutaminase 2 (*GLS2*) results in insufficient production of NADPH, thus hindering the negative feedback for ALA-synthase (ALA-S). Under such conditions, ferrochelatase (FECH) is saturated and hence metabolizes a large amount of exogenous 5-ALA, thus resulting in the temporary accumulation of protoporphyrin IX (PpIX). At the area of no fluorescence intensity (blue area), *GLS2* expression is normal, and sufficient NADPH is produced to support the normal feedback mechanism for ALA-synthase. In this case, adequate FECH activity metabolizes exogenous 5-ALA without delay. *Filled arrowhead*; sufficient amount or activity of molecule or enzyme, *Open arrowhead; insufficient amount or activity of molecule or enzyme, Thick color arrow; smooth metabolic process. Thin color arrow; delayed metabolic process. Dashed red line; abnormal feedback mechanism. Solid blue line; normal feedback mechanism., Dashed arrow; abbreviated metabolic process*.

On the basis of the evidence obtained, we deduced that *GLS2* expression and the intracellular NADPH level are key players in the 5-ALA fluorescence in GBM tissue. There is crosstalk between the TCA cycle and heme synthesis pathway through succinyl coenzyme A (Succinyl-CoA) and glycine to form 5-ALA by ALA synthase^19,26–30^. The rate-limiting step of the heme synthesis pathway is ALA synthase, which is negatively regulated by heme [which is usually degraded by heme oxygenase (HO-1)], via a feedback mechanism^26^. In this step, HO degrades heme in concert with NADPH cytochrome P450 reductase, and it has been shown that HO activity is increased by NADPH or NADH^31^. Therefore, if *GLS2* were underexpressed, NADPH production would be decreased, and insufficient NADPH would result in decreased HO activity, thereby saturating FECH because of heme synthesis pathway over-activation. Thus, exogenous 5-ALA cannot be metabolized rapidly, and PpIX accumulates under such conditions (Fig. 5). However, if *GLS2* expression were normal, there would be sufficient NADPH production to facilitate HO activity in the normal negative feedback mechanism of 5-ALA synthesis. Thus, sufficient FECH activity allows for exogenous 5-ALA to be metabolized without delay (Fig. 5). Therefore, the regional heterogeneity of *GLS2* expression in GBM tissue is responsible for the 5-ALA fluorescence heterogeneity.

## Discussion

Glutamine is an essential fuel source for cancer cell metabolism, which is required for cancer growth and proliferation^32^. Glutamine is converted by glutaminases to ammonium ion and glutamate in the mitochondria, and the glutamate further catabolized to alpha-ketoglutarate (α-KG), which enters the TCA cycle. There are two glutaminase types in mammals, as Kreb had originally described, which are tissue specific^33^. Of the two glutaminase isoforms, *GLS* (kidney-type) is more broadly expressed in normal tissue except for the liver, whereas *GLS2* (liver-type) is exclusively expressed in the liver, brain, and pancreas^34^. Interestingly, evidence has shown that GLS and GLS2 differ in their regulation and activity. GLS is inhibited by its product glutamate, which is not the case with GLS2; however, GLS2, but not GLS, is activated by its product ammonia *in vitro* ^35^. In terms of the oncogenic process, *GLS* expression is regulated by the oncogene *c-myc* ^36^, whereas *GLS2* is induced by the tumor suppressor p53^32,33^. Additionally, GLS is known to have oncogenic properties^36^, whereas GLS2 acts as a tumor suppressor^33^. Silencing *GLS* induces apoptosis in glioma cells, whereas *GLS2* overexpression suppresses malignant properties^37^. Moreover, the switching to GLS upregulation in combination with GLS2 repression in hepatoma cells has been found to be accompanied by malignant transformation in an experimental setting^38^. Cells with elevated *GLS2* expression show increased glutamate concentrations, increased mitochondrial respiration, higher GSH and NADH levels, and decreased reactive oxygen species (ROS) levels^32,33^. Additionally, the knock-down of *GLS2* increases the intracellular ROS levels after radiation by decreasing the production of antioxidant GSH, NADH and NADPH^34^. However, knowledge concerning the role of *GLS2* in cancer is relatively limited compared with that of *GLS*.

There is substantial evidence that glutamine metabolism is crucial in gliomas^39^. The lack of GLS2 expression has been reported in human malignant glioma samples^35^. Additionally, low *GLS2* expression is a specific property of tumor cells of glial origin^39^. Transfection of T98G human GBM cells with *GLS2* decreases cell proliferation, survival, and migration^40^. Moreover, transfection with *GLS2* alters the expression pattern of many genes relevant to malignancy^40^. The opposite effect of *GLS* and *GLS2* on cell growth has also been demonstrated in GBM cells^39^. However, glutamine metabolism is heterogeneous in GBM. There is evidence that glutamine synthetase (GS) activity is variable within the GBM microenvironment in that some cells exhibit high GS expression and glutamine self-sufficiency, whereas others exhibit low GS levels and dependence on exogenous glutamine^41^. Another report has shown striking differences in the glutamate level between GBM and adjacent normal brain tissues, thus supporting regional metabolic heterogeneity of GBMs^42^. The results of the present study are in line with the previously mentioned observations regarding GBM, that are the importance of glutamine metabolism involving *GLS2*, ii) the tumor suppressor effect of *GLS2*, and iii) the regional metabolic heterogeneity in tumor tissues. Considering these findings together, we conclude that regional metabolic heterogeneity in GBM results in low *GLS2* expression in the malignant area with low NADPH and GSH levels (high ROS) and high *GLS2* expression in the lower grade area with high NADPH and GSH levels (low ROS).

In the present study, we propose that NADPH is a metabolic mediator of 5-ALA fluorescence intensity. Both NADPH and GSH were significantly depleted after 5-ALA treatment. However, the NADPH level was increased more than that of GSH after *GLS2* overexpression. Additionally, *in vitro* evidence, as well as human tissue samples of GBM, showed that 5-ALA fluorescence was inversely correlated with GLS2 expression and NADPH level. There is growing evidence in cancer metabolomics highlighting the role of NADPH in oncogenic signaling. The increased demand of NADPH to participate in reductive biosynthesis and redox homeostasis is essential in cancer cells in hypoxic microenvironments^24^. Additionally, there are various mechanisms by which glutamine affects NADPH metabolism and redox control^36^. Canonical NADPH production pathways include a complex source such as the pentose phosphate pathway, serine-glycine metabolism, malic enzyme, IDH, and glutamate dehydrogenase (Supplementary Figure 5A)^43^. These metabolic pathways are frequently altered in cancers. One good example is mutant IDH1 glioma (Supplementary Figure 5B). It has been reported that IDH activity is the main supplier of NADPH in the human brain and glioma, as compared with the rodent brain, and it depends primarily on the pentose phosphate pathway^44^. NADPH production is decreased by more than 40% in gliomas with the IDH1 mutation^38,45^. Our previous study has also shown decreased NADPH production (and increased consumption) in IDH1-mutated malignant gliomas and its implications in 5-ALA fluorescence enhancement^18^. The lack of NADPH inhibits the reduction of GSSG to GSH, which is required for ROS clearance^46^. However, most GBMs express wild-type IDH1, thus suggesting the existence of other major metabolic reprogramming mechanisms influencing NADPH production (Supplementary Figure 5C). To our knowledge, no data on the direct measurement of NADPH in human GBM tissue have been reported. However, on the basis of our results and evidence from other cancers and *in vitro* experiments, GBM tissue is in a harsh microenvironment state of high ROS with an insufficient level of ROS scavengers such as GSH and NADPH. A recent study has shown compensatory upregulation of *IDH1* to meet the NADPH demand in GBMs^45^. The effects of glutamine or glutamate on NADPH production can be conveyed directly by glutamate dehydrogenase or indirectly by glutamine-derived malate^46,47^. Therefore, an aberrant glutamine pathway originating from *GLS2* underexpression is likely to contribute to the NADPH deficit in GBM.

There are controversies in the clinical evidence of the connection between 5-ALA fluorescence and the abnormal status of the TCA cycle in malignant gliomas^18,48^. However, it is accepted that reprogrammed cancer cell metabolism induces the saturation of FECH and PpIX accumulation by activating heme biosynthesis, owing to TCA cycle metabolite accumulation^49^. We suggest another mechanism of aberrant NADPH production and consumption for this crosstalk between the TCA cycle and the heme biosynthesis pathway. As mentioned above, high demand and an insufficient supply of NADPH is the hallmark of the core GBM area, which showed increased 5-ALA fluorescence. Additionally, those areas are under high oxidative stress with increased ROS. Human HO-1 activity has an absolute stoichiometric requirement for NADPH^50^. HO-1 is the rate-limiting enzyme catalyzing the oxidative degradation of cellular heme to liberate free iron. Therefore, the lack of NADPH results in the significant disturbance of the HO-1 activity, which in turn activates the heme biosynthesis pathway, owing to the absence of a feedback mechanism by heme. The result of an overactivated heme biosynthesis pathway decreases the capacity for 5-ALA metabolism. Indeed, a large amount of exogenous 5-ALA administration in cancer cells in this state results in the temporary delay of metabolism and PpIX accumulation, owing to the saturation of FECH. This hypothesis is well correlated with the clinical phenomenon of 5-ALA FGS in which fluorescence exists temporarily in cancer cells between 3 and 9 hours after 5-ALA administration and is washed out completely within a day. The rapid consumption of NADPH and GSH after 5-ALA treatment, as shown in our experiments, also supports their small capacity for metabolism.

In conclusion, although the FGS of malignant glioma using 5-ALA allows for the identification of cancer cells otherwise indistinguishable from normal tissue, there is 5-ALA fluorescence heterogeneity in GBM tissues, which reflects spatial metabolic heterogeneity. This study demonstrated an inverse correlation between the 5-ALA fluorescence intensity and *GLS2* expression in intratumoral tissues of GBM. Moreover, we revealed the metabolic aspects of the mechanism, confirming NADPH as a key molecule in the process. These discoveries improve knowledge about the mechanism of metabolic heterogeneity in GBMs in relation to clinically observable phenomena. NADPH may be developed as a metabolic surrogate marker for the degree of malignancy, given a background of intratumoral heterogeneity. It is expected to contribute to non-invasive molecular diagnostic method development and therapeutic target identification.

## Materials and Methods

### Patient samples

We used prospectively collected samples of 5 histologically verified GBM patients who had undergone FGS for this study. For FGS, 20 mg/kg of 5-ALA (Gliolan^®^; Medac, Wedel, Germany) was administered orally 3–4 hours before anesthesia induction. The main procedure was carried out 5–7 hours after 5-ALA intake. Surgical resection was performed using a Leica M720 OH5 microscope (Leica, Wetzlar, Germany) equipped with an FL400 fluorescence module or a Zeiss Pentero equipped with a fluorescent 400-nm UV light and filters (Zeiss, Oberkochen, Germany). Immediately after the tumor resection, the samples were analyzed for the fluorescence intensity and were classified into 3 groups by using an in-house-devised fluorescence intensity quantification system (see below). The fluorescence intensity was measured on the basis of the red/blue (R/B) ratio in captured images and was divided into the strong (red, R/B ratio >2.0), weak (pink, 0.5 < R/B ratio ≤2.0), and none (blue, R/B ratio ≤0.5) groups. The samples were partly snap-frozen in liquid nitrogen as soon as possible and were stored at −80 °C; a portion of each sample was sent to the pathology department for histological diagnosis.

Another set of samples from 10 GBM patients were collected in the same manner for subsequent NADPH/NADP measurement. This study was performed under the approval of the Institutional Review Board of Seoul National University Hospital, and all experiments were performed in accordance with relevant guidelines and regulations. Written informed consent was obtained from all patients for the usage of samples.

### Fluorescence intensity quantification system

The custom-made fluorescence intensity quantification system was composed of a fluorescence excitation source and a fluorescence image detection system (Fig. 1). Three UV LEDs (405 nm, P8D240, LMH Korea, Hwasung-si, Gyeonggi-do, Korea) were used as a fluorescence excitation source, and SMPS (switched mode power supply) was used as a stable power supplier. The fluorescence image detection system consisted of an optical lens (MNL35M23, Thorlabs, Newton, NJ, USA), an optical long pass filter (450 nm, FEL0450, Thorlabs, Newton, NJ, USA), and a CMOS camera (DCC1645C, Thorlabs, Newton, NJ, USA). The captured images from the fluorescence image detection system were processed to extract fluorescence intensity.

### RNA-sequencing

Before the extraction of RNA, tissues were ground in liquid nitrogen. Total RNA from ground tissues was isolated with an RNeasy Lipid Tissue Mini Kit (Cat No.74804, Qiagen, Hilden, Germany) and was subjected to DNase I treatment (Cat No. 79254, Qiagen, Hilden, Germany) according to the manufacturer’s protocol. The RNA integrity was assessed with a Bioanalyzer system (Agilent, New Haven, CT, USA), and tumor RNAs with an RNA integrity number (RIN) ≥6.5 were subjected to RNA-seq (Supplementary Table 3). RNA-seq libraries were generated using a TruSeq RNA Sample Preparation Kit (Illumina, San Diego, CA, USA). cDNA libraries were sequenced on the HiSeq. 2000 platform (Illumina, San Diego, CA, USA) to obtain approximately 100 million paired-end reads. After removal of poor-quality raw reads containing the adaptor sequence, more than 10% of unknown bases or low-quality bases, the remaining reads were aligned by STAR^51^ to the human reference genome with the GENCODE reference gene annotation version 19. The sequencing reads were counted for genes by using the HTSeq-count^52^. Expression profiles were measured with the unit of FPKM.

### Differentially expressed gene and pathway analysis

After filtering genes with low-sequencing depth across experiments whose mean read counts were less than 20, we statistically evaluated the linear trend of expression levels among the three sample groups to identify differentially expressed genes (FDR < 0.01). Specifically, the generalized linear model that included a patient and sample group as covariates was fitted and statistically tested for the linear trend of gene expression over the ordered factors of sample groups using edgeR^53^. In addition, we required the differentially expressed genes to show fold changes greater than two between groups, to identify genes with a clear linear trend. GSEA was performed for gene set level analysis with the likelihood ratio statistic as an input score^54^.

### Cell culture

Human glioma cell lines T98G, U87MG and LN18 were purchased from the American Type Culture Collection (ATCC, Manassas, VA, USA) and were maintained in Dulbecco’s modified Eagle’s medium (DMEM) supplemented with 10% fetal bovine serum (Gibco, Gran Island, NY, USA), 100 units/ml penicillin and 100 μg/ml streptomycin sulfate (Welgene, Daegu, Korea).

### Gene constructs

Human *GLS2* (GenBank Accession No. NM_013267) cDNA obtained from Origene (Rockville, MD, USA) was cloned into the GenTarget’s expression lentivector. The vector contains a GFP-puromycin dual marker under a Rous sarcoma virus (RSV) promoter, and the cloned *GLS2* was expressed under the enhanced constitutive elongation fator-1 alpha (EF1a) promoter (Supplementary Figure 6).

### Transient transfection and lentiviral transduction

Expression lentiviral particles were produced in 293 T cells in DMEM medium according to the manufacturer’s protocol. The viruses were collected and filtered through 0.45-μm filters. Virus titers were measured via GFP-positive cells after transduction into TH1080 cells; the concentrations of the titers were 1.25 × 10^7^ IFU/mL for Mock and 1.15 × 10^7^ IFU/mL for *GLS2*.

The T98G, U87MG and LN18 cell lines were transduced with the corresponding lentiviruses for 24 hours in the presence of 4 μg/mL polybrene. The transduced cells were selected using puromycin (Cat. No. A7793, Sigma-Aldrich, St Louis, MO, USA), and the transduction efficacy was observed by fluorescence microscopy using green filters (Leica, Wetzlar, Germany). The transduction efficiency of each cell line expressing GFP was over 90%, and then the cells were analyzed using a FACS Calibur cell sorter (BD Bioscience, San Jose, CA, USA) equipped with a 530-nm filter (bandwidth, 15 nm), a 585-nm filter (bandwidth, 21 nm), and Cell-Quest software (BD Bioscience, San Jose, CA).

### Quantitative real-time PCR (qPCR)

For real-time PCR, total RNA from the transduced cells was extracted using a PureLink® RNA Mini Kit (Cat No. 12183018 A. Thermo Fisher Scientific, Waltham, MA, USA). Reverse transcription was performed using 1 μg of total RNA and Maxime RT premix (Cat. No. 25082, iNtRON, Seongnam, Korea). The mRNA level of *GLS2* was determined using TaqMan Gene Expression Assays (Assay ID: Hs00998733_m1 for GLS2 and Hs02786624_g1 for GAPDH) and TaqMan Universal PCR MasterMix (Applied Biosystems, Foster City, CA, USA) according to the manufacturer’s protocol. The relative expression was calculated using the ΔΔC_T_ method and was normalized to GAPDH expression.

### Western blot analysis

Western blot analysis was performed as described previously^55^. In brief, cells were lysed in PRO-PREP™ solution (Cat. No. 17081, iNtRON, Seongnam, Korea) supplemented with protease inhibitors (Roche Applied Science, Indianapolis, IN, USA). Whole cell lysates precleared by centrifugation were used for immunoblotting. Thirty micrograms of each total protein sample was loaded per lane, separated by SDS-PAGE, transferred to a nitrocellulose filter and subjected to immunoblotting with appropriate antibodies. The anti-GAPDH antibody was from Cell Signaling Technology (Cat. No. 2118, Beverly, MA, USA), the anti-glutaminase 2 antibody was from Abcam (Cat. No. ab113509, Cambridge, MA, USA), and HRP-conjugated IgGs were from Jackson ImmunoResearch Laboratories (Cat. No. 111-035-003, West Grove, PA, USA). The bands were detected using a PicoEPD Western Reagent Kit (ELPis Biotech, Daejeon, Korea), and immunoblots were visualized using a ChemiDoc XRS System (Bio-Rad, Redmond, WA, USA).

### *In vitro* 5-ALA treatment

We purchased 5-ALA from Sigma-Aldrich (Cat. No. A7793, St. Louis, MO, USA) and dissolved it in phosphate-buffered saline (PBS, pH 7.4) to make a 500-mM stock solution. Cells were seeded into 24-well plates and then were incubated in 500 μl of medium overnight. After 5-ALA treatment with the indicated concentration for 2 hours, cells were rinsed twice with PBS and then incubated in complete medium until analysis.

### Quantification of PpIX fluorescence

To quantify intracellular PpIX fluorescence, we followed a previously described method with modifications (32). Specifically, after 5-ALA treatment, cells were lysed with 60 μl of 0.2% (vol/vol) Triton X-100 on ice for 5 minutes and were centrifuged at 13,000 rpm for 5 minutes at 4 °C. Next, 5 μl of cell supernatant was transferred and subjected to the BCA Protein Assay, and the results were used to normalize the detected relative fluorescence units (RFUs) of PpIX. To detect PpIX fluorescence, 100 μl of methanolic perchloric acid, consisting of 5.6% (vol/vol) perchloric acid in 50% (vol/vol) methanol, was added to the sample and incubated at 37 °C in the dark for 15 minutes. The sample was centrifuged at 13,000 rpm for 5 minutes at 4 °C. Next, 100 μl of supernatant was transferred into 96-well black plates, and the fluorescence intensity was measured using an Infinite M200 PRO instrument (TECAN, Männedorf, Switzerland) and MagellanTM software at a fluorescence excitation wavelength of 400 ± 9 nm and an emission wavelength of 645 ± 20 nm.

To visualize PpIX fluorescence, cells were fixed with 4% paraformaldehyde in PBS at room temperature for 10 minutes, washed again with PBS twice, and mounted using Slow Fade Gold (Cat No. S36936, Invitrogen, Carlsbad, California, USA). The fluorescence images were acquired under 400-fold magnification using a Leica TCS SP8 X confocal laser scanning microscope (Leica Microsystems GmbH, Mannheim, Germany) equipped with a Leica Plan APO 0.4NA objective lens and a PMT detector. The data were analyzed using Leica LASX software (Leica Microsystems GmbH). PpIX fluorescence was excited at 405 nm, and images were collected in the red channel using a 595-nm long-pass emission filter. Confocal images of PpIX fluorescence were collected with a Leica TCS SP8 X confocal laser-scanning microscope (Leica Microsystems GmbH, Mannheim, Germany) at 1024 × 1024 pixels using a HC PL APO CS 40 × water immersion objective (1.10 NA; Leica). Excitation wavelength of 405 nm (UV diode) and peak emission wavelength of 595 nm were used to observe PPIX fluorescence.

### Metabolite screening via LC-MS

Metabolite extraction was performed on LN18, T98G, U87 cells (3 × 10^6^ cells). The harvested cells were quickly washed with cold PBS and were extracted directly with 500 μL of a cold methanol/ACN/water (5:3:2, v/v) mixture. The samples were centrifuged at 28,000 g for 20 min at 4 °C. The supernatant was collected and dried with a centrifugal vacuum evaporator (Vision, Seoul, Korea), and the pellets were collected for protein quantification with a BCA protein assay kit (Thermo Fisher Scientific, Rockford, IL, USA). The dried extracts were dissolved in 20 μL of a mixture of acetonitrile/water (1:1, v/v) and then were transferred into glass vials with micro-inserts for small-volume injections.

Analysis was carried out using an Agilent 1100 Series liquid chromatography system, and an auto-sampler set at 4 °C (Agilent, Santa Clara, California, USA). Chromatographic separation was performed by injection of 4 μl of the sample on a ZIC-pHILIC Polymeric Beads Peek column (150 × 2.1 mm, 5 μm, Merck kGaA, Darmstadt, Germany) at 35 °C and a 0.15 mL/min flow rate, using 10 mM ammonium carbonate (pH = 9.0) in distilled water as mobile phase A and acetonitrile (ACN) as mobile phase B. The linear gradient was as follows: 80% B at 0 minutes, 35% B at 10 minutes, 5% B at 12 minutes, 5% B at 25 minutes, 80% B at 25.1 minutes, and 80% B at 35 minutes.

During the LC-MS experiments, mass spectra were acquired in negative ion mode using an API 2000 Mass Spectrometer (AB/SCIEX, Framingham, MA, USA) equipped with an electrospray ionization (ESI) source. The parameters of the ESI source operation were as follows: −4.5 kV of ion spray voltage; curtain gas (nitrogen), ion source gas 1 (nitrogen), and ion source gas 2 (nitrogen) pressures at 15, 70, and 90, respectively; temperature of the heater (turbo) gas at 550 °C. For the detection, MRM was performed with the m/z values of the precursor and fragment ions as indicated in the Supplementary Table 4, which were established with standard compounds. MRM was performed and controlled with Analyst 1.6 Software. Chromatographic peaks were identified by comparing the retention times, m/z values of the parent and daughter ions with those in the HMDB (www.hmdb.ca), METLIN (https://metlin.scripps.edu) and Massbank (www.massbank.jp) databases and those of the standards.

### NADPH and NADP measurement

The ratios of NADPH/NADP + in the GBM cell lines were calculated using the NADP/NADPH Quantification Kit (BioVision, Mountain View, CA, USA) according to the manufacturer’s instructions. In brief, cell pellets were lysed in extraction buffer with vigorous shaking in an automatic homogenizer (Automill™; Tokken, Chiba, Japan). After centrifugation, the supernatant was filtered through a 10-kDa spin column (Cat. No. ab113509, Abcam, Cambridge, MA, USA) to remove NADPH-consuming enzymes. To detect the total NADPH/NADP + (NADPt), 50 μl of the extracted supernatant was placed into 96-well plates. To detect NADPH only, heat was applied to the supernatant sample at 60°C for 30 min in a heating block (Throughout this process, all NADP + was decomposed, whereas the NADPH remained intact). Then, 50 μl of NADP-decomposed NADPH samples were added to 96-well plates. Next, 100 μl of the cycling mixture was also added to each well, and the plate was incubated for 5 min to convert NADP to NADPH. Ten microliters of NADPH developer was continuously added, and the plates were incubated for 1 hour. Finally, the plates were read at 450 nm by using a microplate reader (Bio-Rad, Hercules, CA, USA).

### Statistical Analyses

All statistical analyses were performed using a free statistical software package, R (version 3.0.2; http://www.r-project.org/), and IBM SPSS Statistics software (version 21; SPSS, Chicago, IL, USA). The data were expressed as the means ± SEM. Student’s t-test was used for the comparison of mean values. Statistical significance was accepted at a level of p < 0.05 (*).

## Electronic supplementary material

Supplementary figures and tables
