# Supplementary material for: Glutaminase 2 expression is associated with regional heterogeneity of 5-aminolevulinic acid fluorescence in glioblastoma
Source: Sci Rep. 2017 Sep 22;7:12221. doi: 10.1038/s41598-017-12557-3 (PMC5610329; doi:10.1038/s41598-017-12557-3)
Supplement: Supplementary file 1 — Supplementary figures and tables [file 41598_2017_12557_MOESM1_ESM.pdf]

Title: Glutaminase 2 expression is associated with regional heterogeneity of 5-aminolevulinic acid fluorescence in glioblastoma

Authors: †Sojin Kim[1], †Ja Eun Kim[2], †Yong Hwy Kim[1], †Taeyoung Hwang[3], Sung Kwon Kim[4], Wen Jun Xu[5], Jong-Yeon Shin[6], Jong-Il Kim[7], Hyoungseon Choi[8], Hee Chan Kim[9], Hye Rim Cho[10], Anna Choi[1], Tamrin Chowdury[1], Youngbeom Seo[11], Yun-Sik Dho[1], Jin Wook Kim[1], Dong Gyu Kim[1], Sung-Hye Park[12], Hyeonjin Kim[10], Seung Hong Choi[10], Sunghyouk Park[5], Se-Hoon Lee[13], Chul-Kee Park[1*]

†These authors contributed equally to this work.

[1]Department of Neurosurgery, Seoul National University College of Medicine, Seoul National University Hospital, Seoul, Korea
[2]Cell and Gene Therapy Products Division, National Institute of Food and Drug Safety Evaluation, Ministry of Food and Drug Safety, Cheongju, Korea
[3]Lieber Institute for Brain Development, Johns Hopkins Medical Campus, Baltimore, MD, USA
[4]Department of Neurosurgery, Gyeongsang National University School of Medicine, Gyeongsang National University Hospital, Jinju, Korea
[5]College of Pharmacy, Natural Product Research Institute, Seoul National University, Seoul, Korea
[6]Genomic Medicine Institute, Medical Research Center, Seoul National University, Seoul, Korea
[7]Department of Biochemical and Molecular Biology, Seoul National University College of Medicine, Seoul, Korea
[8]DMC R&D center, Samsung Electronics Co., Ltd., Seoul, Korea
[9]Department of Biomedical Engineering, College of Medicine and Institute of Medical and Biological Engineering, Medical Research Center, Seoul National University, Seoul, Korea
[10]Department of Radiology, Seoul National University College of Medicine, Seoul National University Hospital, Seoul, Korea
[11]Department of Neurosurgery, Yeungnam University College of Medicine, Daegu, Korea
[12]Department of Pathology, Seoul National University College of Medicine, Seoul National University Hospital, Seoul, Korea
[13]Division of Hematology-Oncology, Department of Medicine, Samsung Medical Center, Sungkyunkwan University School of Medicine, Seoul, Korea

Supplements

Supplementary Figure 1

Supplementary Figure 2

Supplementary Figure 3

Supplementary Figure 4

Supplementary Figure 5

Supplementary Figure 6

Supplementary Table 1

Supplementary Table 2

Supplementary Table 3

Supplementary Table 4

Supplementary Figure 1. Intraoperative picture of tumors of all 5 glioblastoma cases showing heterogeneous fluorescence under illumination with ultrawave light of 440-nm wavelength after 5-aminolevulinic acid (5-ALA) administration.

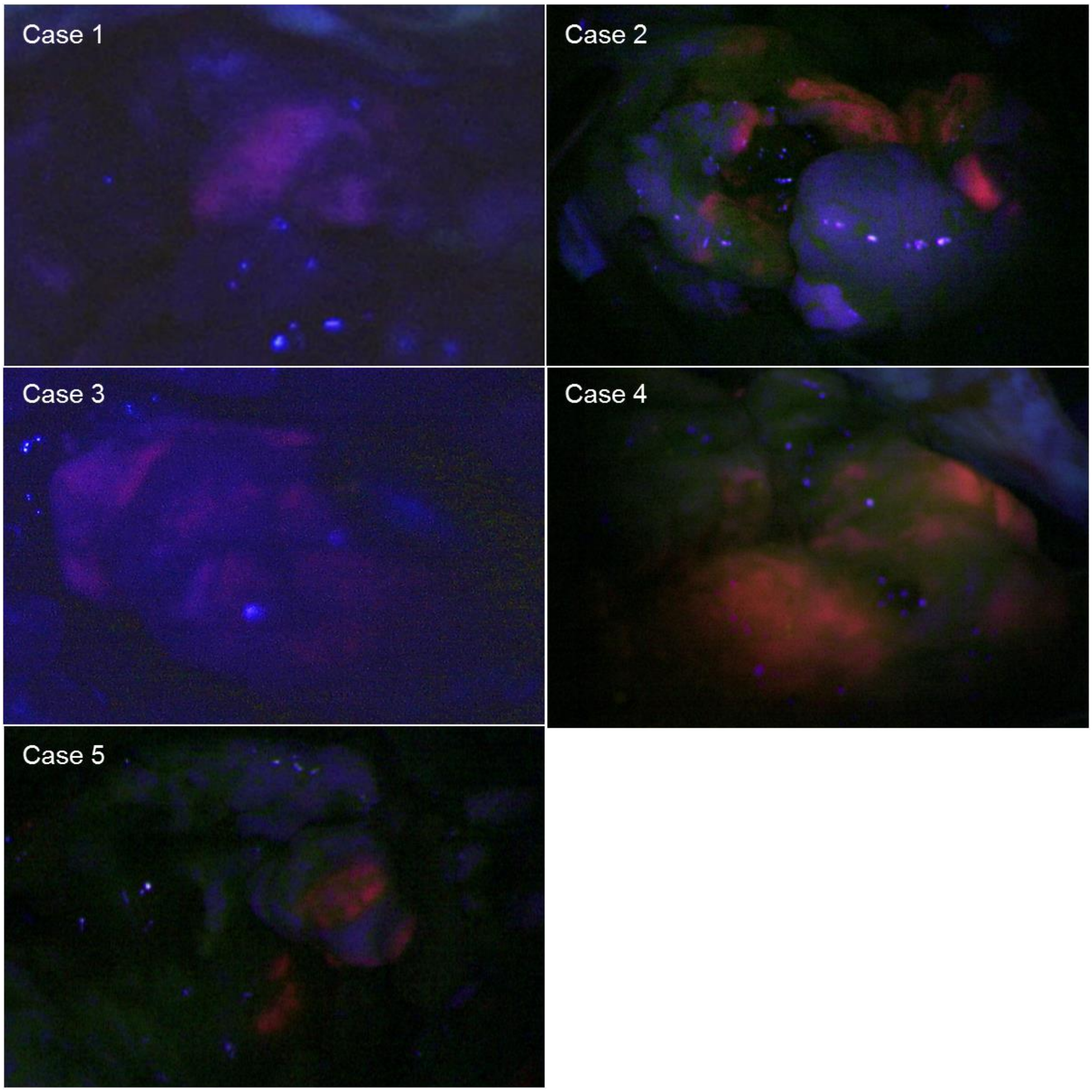

Supplementary Figure 2. Histological features of area with different 5-ALA fluorescence. Area of red fluorescence showed features of definitive grade IV glioblastoma. Area of pink fluorescence showed lower grade features than area of red fluorescence. Area of blue (no fluorescence) showed low grade features or only reactive gliosis in all cases.

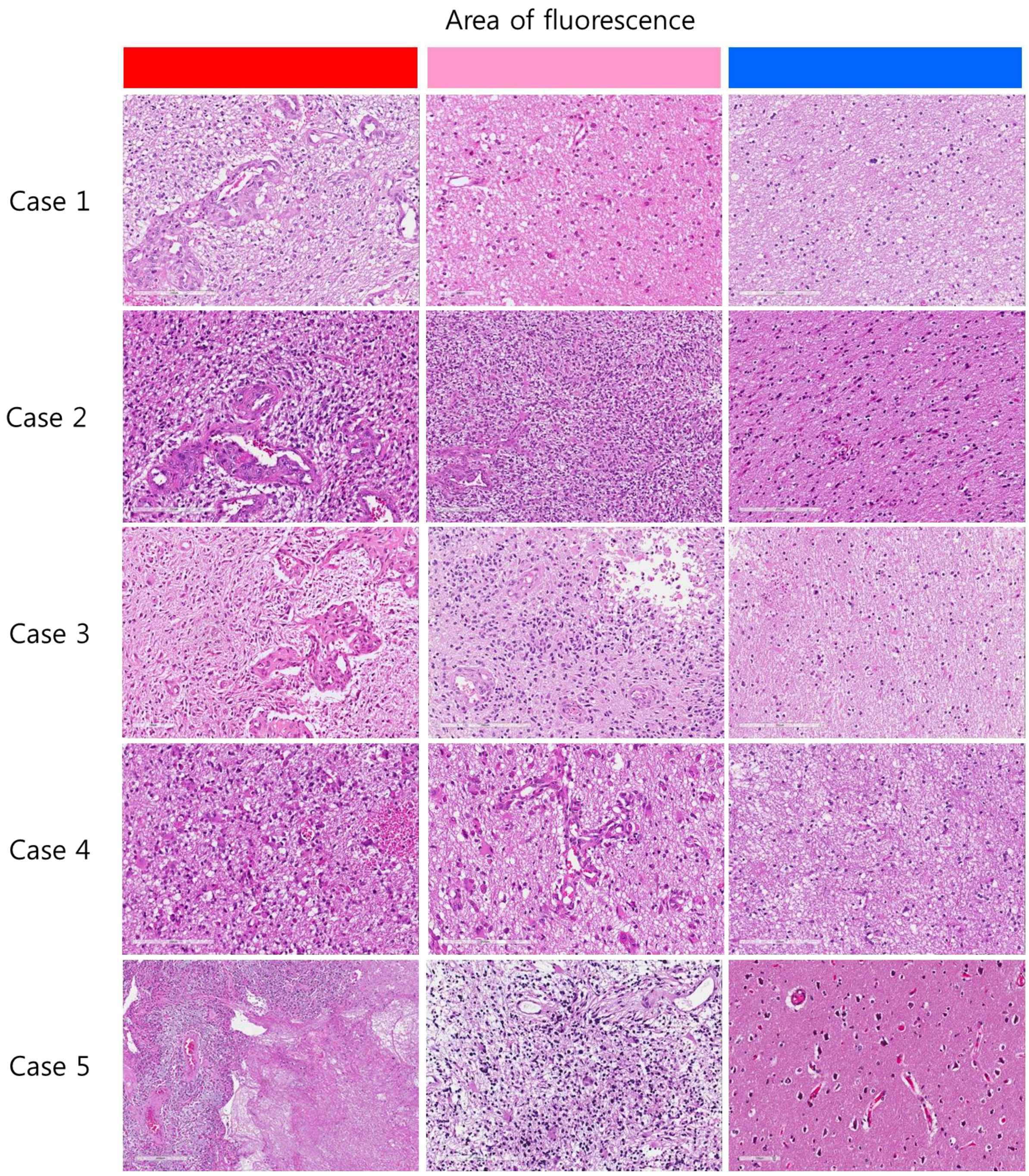

Supplementary Figure 3. Full-length gel images of western blot used in figure 3A. There was and no manipulation of images after acquisition after 10 seconds of exposure time.

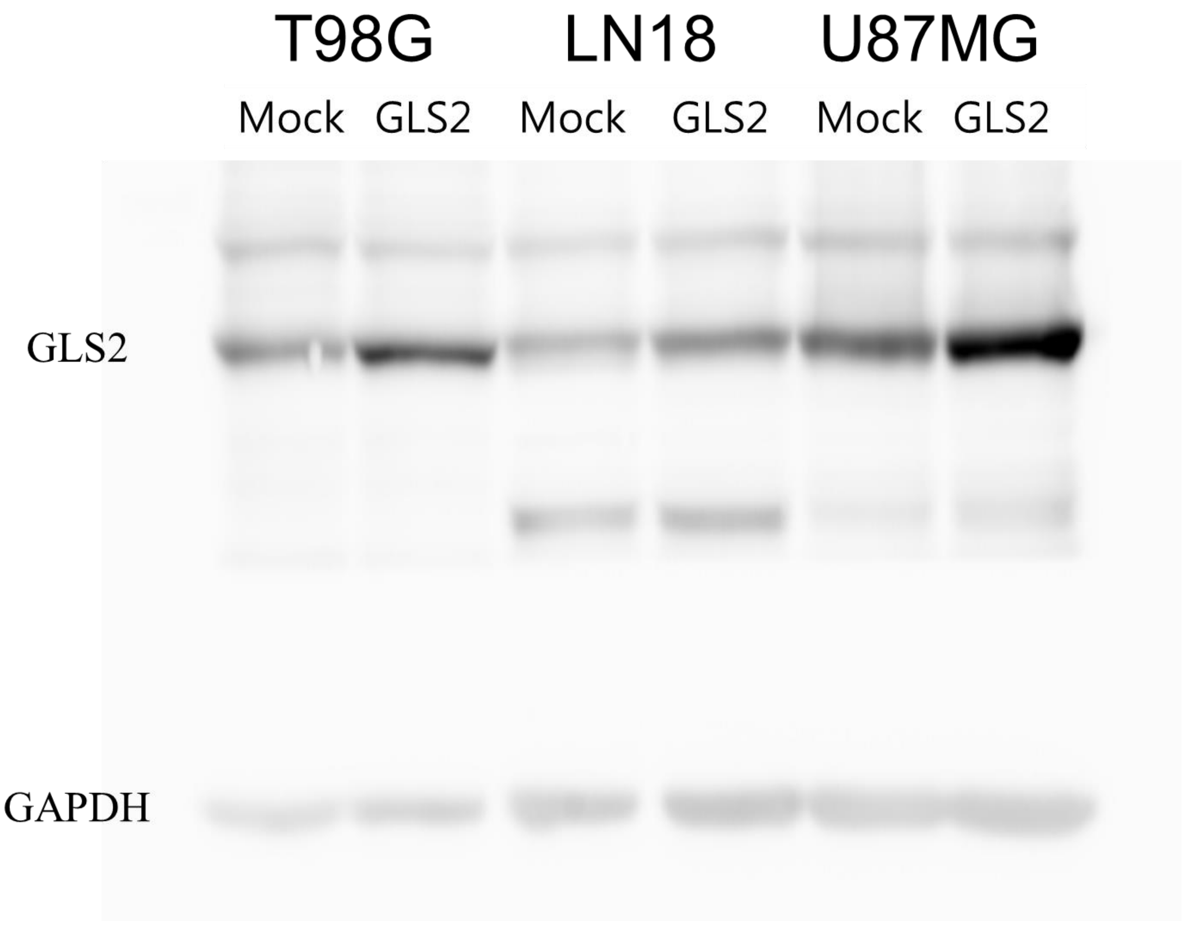

Supplementary Figure 4. Energy metabolism changes regarding to *GLS2* expression or 5-ALA treatment represented by AMP, ADP, and ATP levels.

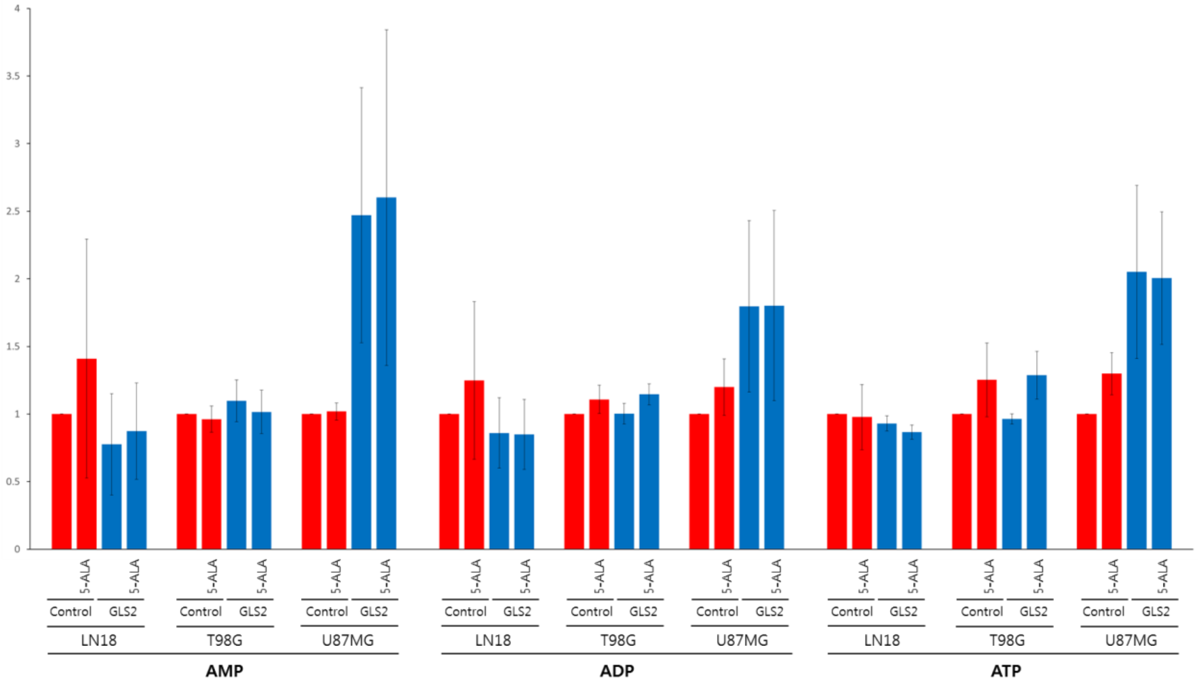

Supplementary Figure 5. A. Canonical pathways of NADPH production in normal state of the cells. Produced NADPHs are used to clear the reactive oxygen species (ROS) by reducing GSSG to GSH. **B.** In IDH1 mutated gliomas, NADPH production is decreased at the stage of isocitrate to a-KG conversion, and NADPH is rapidly consumed by producing oncometabolite 2-HG. **C**. In glioblastoma, underexpresison of GLS2 is the main reason for the decreased production of NADPH as described in the present study.

A.

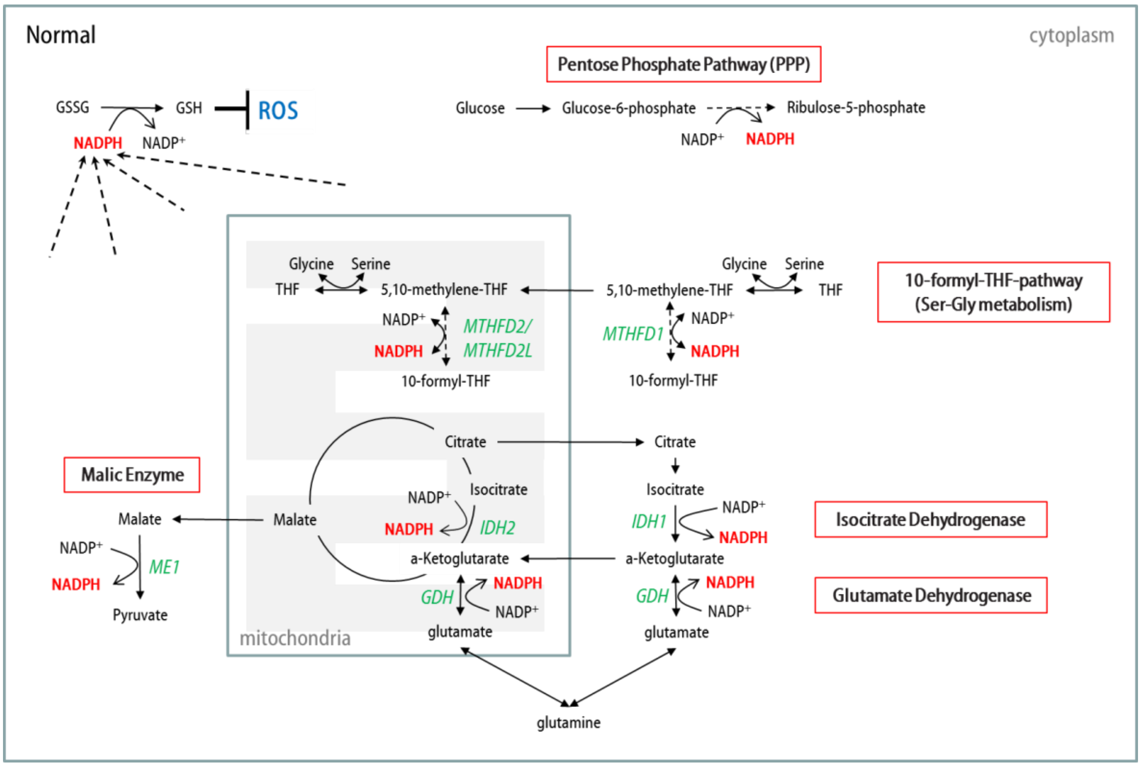

B.

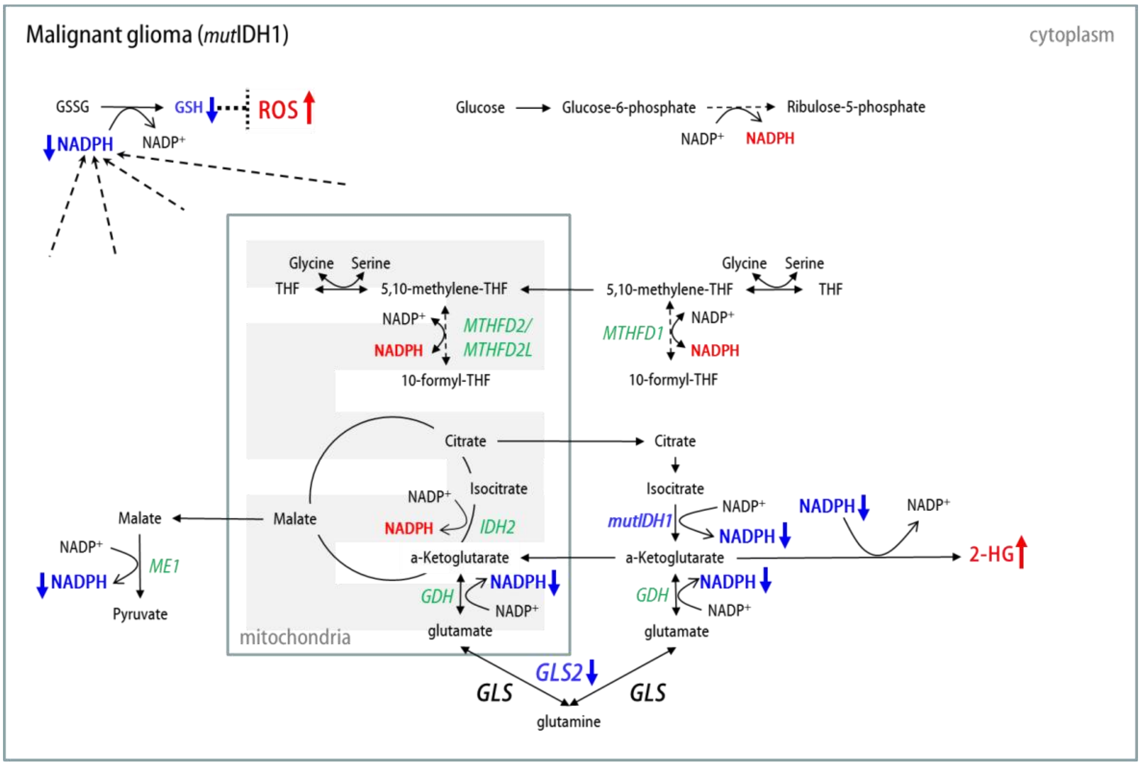
Malignant glioma (mutIDH1)
cytoplasm
GSSG
GSH
ROS
NADPH
NADP+
Glucose
Glucose-6-phosphate
Ribulose-5-phosphate
NADP+
NADPH
Glycine
Serine
THF
5,10-methylene-THF
NADP+
NADPH
MTHFD2/
MTHFD2L
10-formyl-THF
Glycine
Serine
5,10-methylene-THF
THF
MTHFD1
NADP+
NADPH
10-formyl-THF
Citrate
Citrate
Isocitrate
NADP+
Isocitrate
Malate
Malate
NADPH
IDH2
mutIDH1
NADP+
NADPH
NADPH
NADP+
2-HG
NADP+
ME1
NADPH
Pyruvate
a-Ketoglutarate
a-Ketoglutarate
GDH
NADPH
NADP+
GDH
NADPH
NADP+
glutamate
glutamate
mitochondria
GLS2
GLS
GLS
glutamine

C.

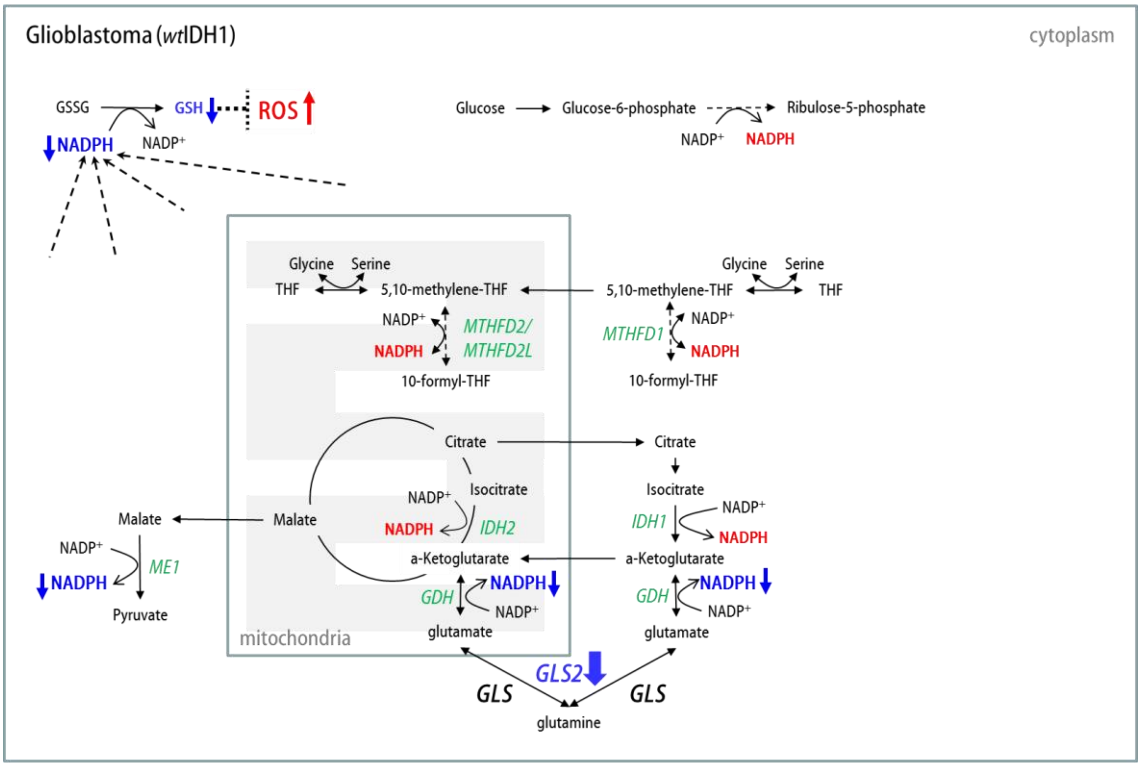
Glioblastoma (wtIDH1)
cytoplasm
GSSG
GSH
ROS
NADPH
NADP+
Glucose
Glucose-6-phosphate
Ribulose-5-phosphate
NADP+
NADPH
Glycine
Serine
THF
5,10-methylene-THF
NADP+
NADPH
MTHFD2/
MTHFD2L
10-formyl-THF
Glycine
Serine
5,10-methylene-THF
THF
MTHFD1
NADP+
NADPH
10-formyl-THF
Citrate
Citrate
Isocitrate
NADP+
Isocitrate
Malate
Malate
NADPH
IDH2
IDH1
NADP+
NADPH
NADP+
ME1
NADPH
Pyruvate
a-Ketoglutarate
a-Ketoglutarate
GDH
NADPH
NADP+
GDH
NADPH
NADP+
glutamate
glutamate
mitochondria
GLS2
GLS
GLS
glutamine

Supplementary Figure 6. Structure of the lentiviral GLS2 gene construct.

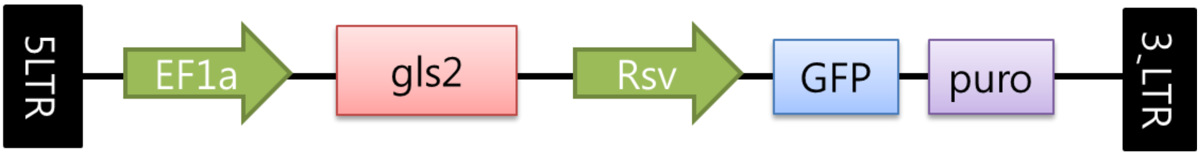

Supplementary Table 1. Fluorescence intensity of collected samples expressed by red/blue (R/B) ratio.

Patients	samples	R/B ratio
Case 1	Red	2.3
	Pink	0.99
	Blue	0.25
Case 2	Red	2.45
	Pink	0.88
	Blue	0.27
Case 3	Red	2.12
	Pink	1.06
	Blue	0.31
Case 4	Red	2.31
	Pink	1.18
	Blue	0.36
Case 5	Red	2.51
	Pink	1.13
	Blue	0.13

Supplementary Table 2. Significantly enriched gene sets resulted from GSEA preranked using differentially expressed genes along with fluorescence intensity (p < 0.01 and FDR < 0.25).

Gene set	Size	ES	NES	NOM p-val	FDR q-val	FWER p-val
KEGG_TASTE_TRANSDUCTION	24	0.802278	1.421605	0.001	0.041006	0.039
KEGG_NITROGEN_METABOLISM	19	0.776089	1.37556	0.00201	0.074512	0.136
KEGG_NEUROACTIVE_LIGAND_RECEPTOR_INTERACTIO N	188	0.700356	1.336668	0	0.136354	0.34
KEGG_CALCIUM_SIGNALING_PATHWAY	157	0.698186	1.331653	0	0.11377	0.371
KEGG_LONG_TERM_POTENTIATION	64	0.71273	1.322066	0	0.111221	0.426
KEGG_TYPE_II_DIABETES_MELLITUS	40	0.716111	1.308326	0.002	0.108304	0.529
KEGG_ABC_TRANSPORTERS	40	0.709466	1.299255	0.002	0.117142	0.603
KEGG_AMYOTROPHIC_LATERAL_SCLEROSIS_ALS	51	0.688828	1.276808	0.003	0.149722	0.783
KEGG_PHOSPHATIDYLINOSITOL_SIGNALING_SYSTEM	71	0.678931	1.26703	0	0.163297	0.846
KEGG_CARDIAC_MUSCLE_CONTRACTION	60	0.679627	1.261082	0	0.166857	0.877
REACTOME_BOTULINUM_NEUROTOXICITY	18	0.870928	1.530712	0	0.00296	0.003
REACTOME_GLUTAMATE_NEUROTRANSMITTER_RELEA SE_CYCLE	15	0.87877	1.513251	0	0.003205	0.013
REACTOME_NEUROTRANSMITTER_RELEASE_CYCLE	30	0.834111	1.505116	0	0.003286	0.02
REACTOME_GABA_SYNTHESIS_RELEASE_REUPTAKE_A ND_DEGRADATION	17	0.879336	1.51914	0	0.003453	0.007
REACTOME_PROTEOLYTIC_CLEAVAGE_OF_SNARE_COM PLEX_PROTEINS	16	0.871566	1.515746	0	0.003616	0.011
REACTOME_INTERACTION_BETWEEN_L1_AND_ANKYRI NS	19	0.861466	1.50729	0	0.003746	0.019
REACTOME_LIGAND_GATED_ION_CHANNEL_TRANSPO RT	15	0.856983	1.481922	0	0.007181	0.051
REACTOME_RAS_ACTIVATION_UOPN_CA2_INFUX_THRO UGH_NMDA_RECEPTOR	17	0.811236	1.447702	0.001001	0.017497	0.133
REACTOME_GABA_RECEPTOR_ACTIVATION	46	0.780473	1.436715	0	0.02081	0.174
REACTOME_NEURONAL_SYSTEM	251	0.737683	1.417356	0	0.026862	0.287
REACTOME_ION_CHANNEL_TRANSPORT	44	0.773427	1.413555	0	0.027829	0.313
REACTOME_TRAFFICKING_OF_GLUR2_CONTAINING_A MPA_RECEPTORS	15	0.816335	1.418835	0.005015	0.028049	0.276
REACTOME_TRANSMISSION_ACROSS_CHEMICAL_SYNA PSES	169	0.74386	1.421099	0	0.028981	0.26
REACTOME_AMINE_LIGAND_BINDING_RECEPTORS	28	0.77956	1.40399	0	0.031281	0.38
REACTOME_REGULATION_OF_INSULIN_SECRETION_BY _GLUCAGON_LIKE_PEPTIDE1	37	0.767714	1.405709	0	0.032108	0.369
REACTOME_NEUROTRANSMITTER_RECEPTOR_BINDING _AND_ DOWNSTREAM_TRANSMISSION_IN_THE_POSTSYNAPTI C_CELL	125	0.734625	1.393157	0	0.037273	0.447
REACTOME_G_PROTEIN_ACTIVATION	22	0.775936	1.383845	0.003	0.044359	0.536
REACTOME_UNBLOCKING_OF_NMDA_RECEPTOR_GLUT AMATE_BINDING_AND_ACTIVATION	15	0.794122	1.381506	0.012012	0.044523	0.559
REACTOME_TRAFFICKING_OF_AMPA_RECEPTORS	27	0.764779	1.375103	0.001	0.045542	0.6
REACTOME_POTASSIUM_CHANNELS	87	0.732118	1.375815	0	0.047368	0.597

Supplementary Table 3. Summary of RNA-seq quality control.

Patients	samples	RNA integrity number	Total reads	Total yield	Read length	Mappable reads	Mappable yield	%Mappable reads
Case 1	Red	9.0	97,850,132	9,882,863,332	101	86,176,029	8,704,081,929	88.07
	Pink	8.5	98,514,024	9,949,916,424	101	86,940,647	8,781,005,347	88.25
	Blue	6.9	104,983,980	10,603,381,980	101	92,890,316	9,381,921,916	88.48
Case 2	Red	9.1	104,607,786	10,565,386,386	101	92,317,575	9,324,075,075	88.25
	Pink	8.3	101,594,586	10,261,053,186	101	89,584,893	9,048,074,193	88.18
	Blue	7.0	113,779,730	11,491,752,730	101	100,476,997	10,148,176,697	88.31
Case 3	Red	7.0	106,824,230	10,789,247,230	101	93,654,042	9,459,058,242	87.67
	Pink	8.9	99,179,694	10,017,149,094	101	87,165,392	8,803,704,592	87.89
	Blue	8.6	101,816,284	10,283,444,684	101	89,466,746	9,036,141,346	87.87
Case 4	Red	9.3	93,835,670	9,477,402,670	101	82,386,522	8,321,038,722	87.80
	Pink	8.2	95,081,518	9,603,233,318	101	83,488,732	8,432,361,932	87.81
	Blue	7.5	94,673,036	9,561,976,636	101	83,502,148	8,433,716,948	88.20
Case 5	Red	8.8	113,077,766	11,420,854,366	101	99,547,333	10,054,280,633	88.03
	Pink	7.8	129,859,040	13,115,763,040	101	114,152,855	11,529,438,355	87.91
	Blue	6.9	126,506,286	12,777,134,886	101	111,619,355	11,273,554,855	88.23

Supplementary Table 4. MRM parameters for the metabolite detection using LC-MS. The metabolites, mass-to-charge ratio of precursor and fragment ion, and the detection parameters are indicated.

Metabolites	Precursor Ion (m/z)	Fragment Ion (m/z)	DP† (V)	FP† (V)	EP† (V)	CE† (V)	CXP† (V)
NADPH	744.198	78.9	-36	-50	-10.5	-128	-4
NADP	742.206	620.1	-16	-50	-7	-30	-18
NAD	662.2	540	-27	-50	-6	-26	-20
NADH	663.9	78.9	-31	-50	-11.5	-118	-4
GSH	306	272	-26	-50	-5.5	-16	-16
GSSG	611	306	-31	-50	-10.5	-34	-14
ADP	425.8	79	-31	-50	-12	-108	-4
AMP	345.6	79	-31	-50	-11	-112	-4
ATP	505.8	79	-32	-50	-6	-108	-4
Glutamate	145.809	102.1	-21	-130	-4.5	-20	-4
Glutamine	144.828	126.9	-21	-210	-4	-16	-24

† DP, declustering potential; FP, focusing potential; EP, entrance potential; CE, collision energy; CXP,
